# Petersen's Hernia After Roux-en-Y Gastric Bypass: A Case Report

**DOI:** 10.7759/cureus.50757

**Published:** 2023-12-18

**Authors:** Ibrahim El Nogoomi, Abdelrahman K Nouh, Abdallah A Jaber, Abduljaleel M Toubah, Sana S Alkaram

**Affiliations:** 1 Department of General Surgery, Al Kuwait Hospital, Sharjah, ARE

**Keywords:** bowel obstruction, internal hernia, bariatric surgery, roux-en-y gastric bypass, peterson’s hernia

## Abstract

Internal hernia (IH) is the protrusion of abdominal contents, mostly small bowel loops, through a defect in the peritoneum or mesentery. Petersen’s hernia is a type of internal hernia, in which part of the intestinal loop protrudes through a defect between small bowel limbs, transverse mesocolon, and retroperitoneum. It has been reported in individuals undergoing gastrojejunostomy (GJ), especially following bariatric surgeries. Because of the expanding popularity of these surgical treatments, the total incidence of internal hernias has recently increased. The laparoscopic Roux-en-Y gastric bypass (RYGB) has been proven to be a safe and successful alternative to the classic open RYGB. Although the absence of postoperative adhesions is one advantage of minimally invasive surgery, it facilitates the occurrence of internal hernia with reported rates of 5% three months to three years following surgery. Clinical findings are vague and can vary from mild to severe abdominal pain that can be accompanied by vomiting, nausea, and abdominal distention.

## Introduction

Petersen’s hernia is a type of internal hernia first described by the German surgeon Walther Peterson. This type of hernia occurs when part of the intestinal loop protrudes through a defect between the small bowel limbs, transverse mesocolon, and retroperitoneum. Up until 1974, only 178 incidents were documented. However, with the exponential rise in laparoscopic gastric bypass for the treatment of obesity over the past few years, it has become more common [1], with an estimated incidence that ranges from 0.9% to 4.5% [2].

Internal hernias can have a wide variety of manifestations, ranging from mild abdominal discomfort to severe abdominal pain that resembles small bowel obstruction. The severity of symptoms is determined by the duration, reducibility of the bowel segment, and the presence or absence of strangulation and incarceration [3]. Although an internal hernia is a rare complication of gastrojejunostomy, it accounts for 5.8% of all small bowel obstructions [4]. Rapid detection of such conditions is life-saving, as it can cause potentially fatal consequences that require emergency surgery if left undiagnosed. 

CT scan is becoming more popular in the preoperative workup of internal hernias. Although it has low sensitivity (28%), its high specificity (90%) and the ability to diagnose other pathologies makes it the most recommended imaging modality. Nevertheless, exploratory surgery is the best diagnostic and therapeutic tool [5]. 

In this case report, we describe a case of strangulated Petersen-space hernia after Roux-en-Y gastric bypass.

## Case presentation

A 44-year-old female, with a BMI of 43 kg/m^2^ presented to the emergency department complaining of severe abdominal pain for four days. The pain was localized in the epigastric area and does not radiate elsewhere. The patient described it as intense colicky pain that started gradually and was progressively getting worse, with a score of 7/10 in severity. It got aggravated after eating food with no alleviating factors. The pain was associated with nausea and multiple episodes of non-bilious, clear vomiting. There was no history of fever, trauma, urinary symptoms, or changes in bowel habits.

Apart from a history of toxic goiter in 2004 that was managed and cured then, she had no other medical or family history. In 2014, she underwent laparoscopic sleeve gastrectomy, and two months prior to presentation, she underwent a redo laparoscopic Roux-en-Y gastric bypass due to weight regain. Both surgeries were uncomplicated and had an uneventful hospital course.

On physical examination, she had significant epigastric tenderness and guarding was noted with no other positive signs. She was vitally stable (temperature (T): 36.5°C tympanic, heart rate (HR): 70 bpm, respiratory rate (RR): 16 brpm, BP: 110/69 mmHg, saturation of peripheral oxygen (SpO_2_): 100%) with non-specific laboratory findings and slight elevation of the inflammatory status (WBC: 6790/μl; C-reactive protein (CRP): 4.3 mg/L­; aspartate aminotransferase (AST): 13 IU/L; alanine transaminase (ALT): 17 IU/L; amylase (AMY): 48 IU/L).

**Figure 1:**
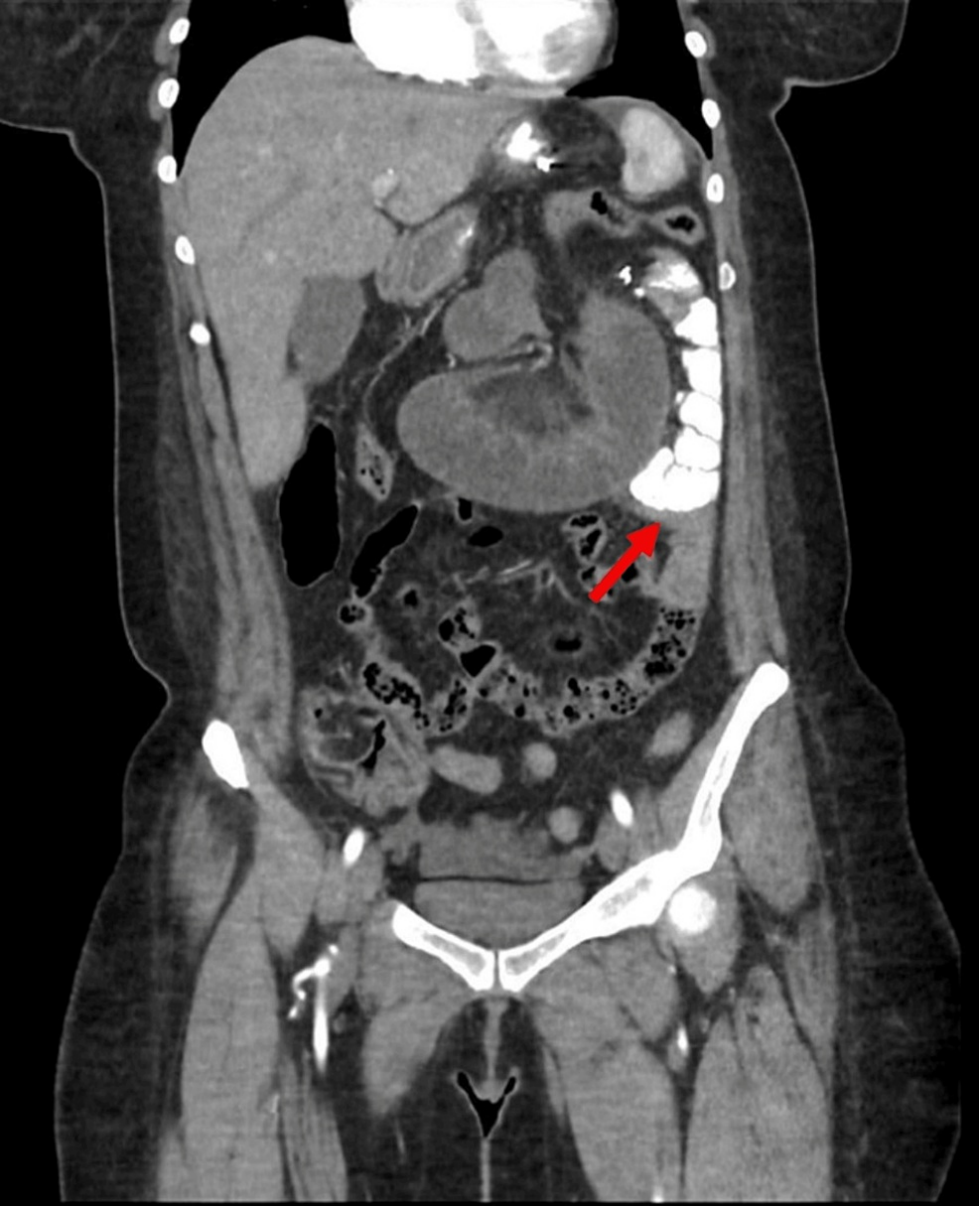
CT abdomen with contrast (coronal view) Showing signs of small bowel obstruction due to improper filling of the contrast, marked dilatation of parts of the small bowel surrounded by effusion

**Figure 2:**
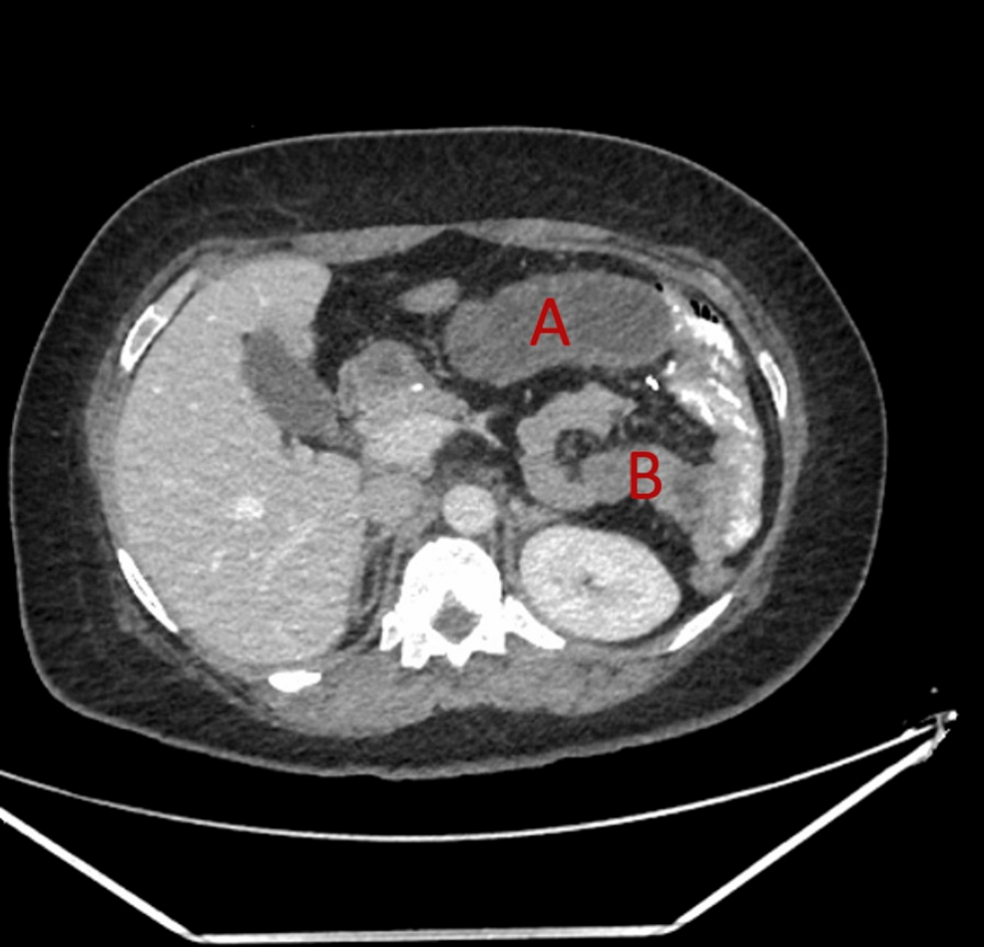
CT abdomen with contrast (axial view) showing the difference between (A) the proximal dilated part of the small intestine and (B) the distal collapsed slightly enhanced part (indicating signs of ischemia)

An abdominal X-ray was done upon the patient's arrival where it was unremarkable. An abdominal CT scan (Figures 1, 2) with oral contrast revealed no extraluminal leakage. A few distended jejunal loops at the left upper abdomen were noted with mild congestion of the mesentery along the transition between the dilated and normal bowel with no other remarkable findings. All of these along with clinical correlation are suggestive of closed-loop obstruction due to internal hernia or adhesion. 

**Figure 3:**
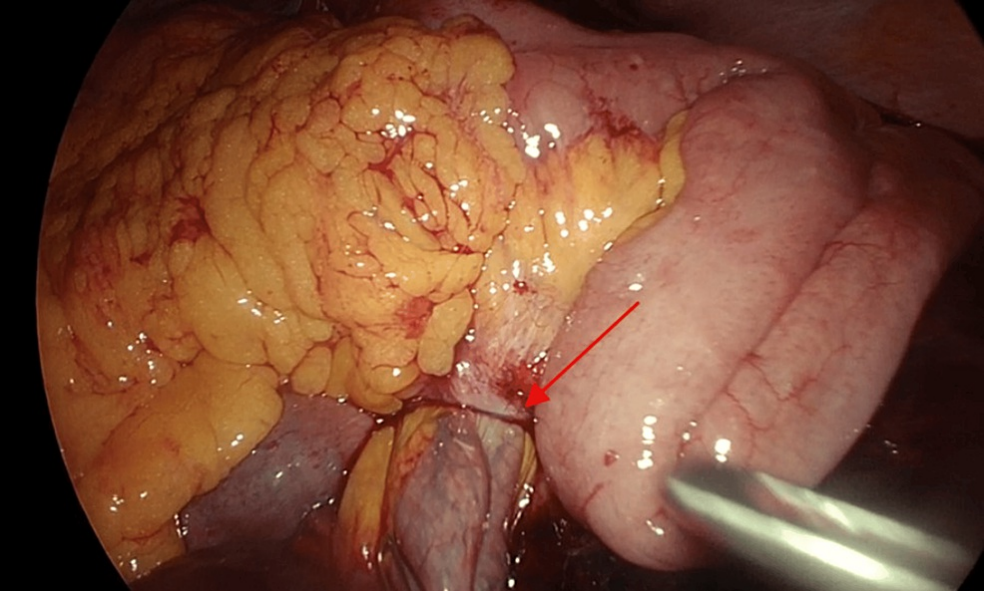
Intraoperative laparoscopic view Showing part of the jejunum protruding through Petersen's defect

The patient underwent diagnostic laparoscopy (Video 1) and was found to have: 1) a strangulated loop of the small bowel herniated from the left side to the right side through the Petersen defect, causing closed-loop obstruction and strangulation through a narrow stitched defect at the primary surgery (Figure 3) and 2) a gangrenous loop of the small bowel about 50 cm long and starting about 25 cm from the duodenojejunal (DJ) junction. Reduction by pulling the strangulated loop from the Petersen defect was done, followed by resection of the gangrenous loop using a 60 mm tri stapler. In addition, mesenteric dissection and resection with Ligasure, followed by a side-to-side anastomosis between the two ends with a 45 mm gold trip stapler and the closure of the defect with a 2 V-Loc suture. The operation ended with a peritoneal lavage leaving two corrugated drains.

**Video 1:** Intraoperative video Showing part of the small bowel protruding through Petersen's space. A gangrenous loop of the small bowel was reduced by pulling the strangulated segment from the Petersen defect.

## Discussion

In our case, we observed a complication after the laparoscopic Roux-en-Y gastric bypass surgery. This led to internal herniation through Petersen's space, causing a closed-loop bowel obstruction. Petersen’s hernia occurs when the bowel herniates through the opening between the jejunal mesentery and the transverse mesocolon. These internal hernias can happen in around 7% of cases if the defects are not repaired [6]. Internal herniation is naturally prevented by the transverse colon and mesocolon, which serve as a barrier between the stomach and the small intestine. Once a connection is made between the stomach and small intestine through any type of gastrojejunal anastomosis, a space is formed behind the small intestine's limbs, potentially allowing for internal herniation. Another factor that plays a role in developing an internal hernia is that as patients lose weight, bowel anatomy undergoes changes, and any existing defects with the mesentery can become more pronounced, or new ones can arise [1].

The diagnosis of internal hernia is challenging, the reason behind that is the vague and nonspecific clinical presentation and the lack of a sensitive diagnostic test that can be used for screening. Thus, keeping a high clinical suspicion is crucial to avoid delay in diagnosis or misdiagnosis. The clinical presentation of internal hernia can vary from vague mild abdominal discomfort to severe abdominal pain. The severity of symptoms depends on 1) the duration of the hernia, 2) the reducibility of the hernia, and 3) the presence of strangulation or incarceration [3]. The most common presentation of internal hernia is strangulating small bowel obstruction. However, it can remain clinically silent, especially those small easily reducible hernias in comparison to the large hernias that can be more critical. A potentially fatal condition occurs when the intestinal loop gets incarcerated, which results in bowel obstruction and strangulation, and if not treated urgently, can result in necrosis of the affected bowel loop.

Computed tomography (CT) plays a major role in the diagnostic workup of internal hernias. Since time is very sensitive in such cases and the interval between bowel obstruction and strangulation can be short, the need for a fast and accurate diagnostic test is essential. Several studies have shown the accuracy of computed tomography in the diagnosis of small bowel obstruction with a sensitivity and specificity of 94-100% and 90-95% respectively. One study has suggested that computed tomography is not only accurate in diagnosing internal hernias but even in determining their location [7]. Small bowel obstruction caused by an internal hernia is usually visualized as a closed-loop obstruction on computed tomography, in which the small bowel is obstructed at a proximal and a distal point, making a U- or C-shaped distended loop that is filled with fluid. In addition, the hernia orifice is lined up with the colon, mesenteric fat, and closed-loop arteries while the surrounding tissue and major blood vessels are abnormally displaced around the hernia sac.

Although laparoscopic surgery has many benefits, the rising popularity of laparoscopic Roux-en-Y gastric bypass for treating severe obesity has resulted in a growing number of cases reporting small bowel obstruction caused by internal hernias. This is due to the fact that after laparoscopic gastric surgery, the small bowel becomes more mobile due to fewer adhesions, which can lead to internal hernias [1]. In addition, the antecolic route is preferably used against its counterpart - the retrocolic route - for having better exposure to the abdominal anatomical defects (mesenteric and lesser mesenteric defects). However, some studies concluded that using this route was found to increase the incidence of future internal hernia, especially through the Petersen defect [1].

There have been several methods to prevent such complications during/after ante-colic Roux-en-Y reconstruction. A method that is popularly used by surgeons is the closure of the defects, thus decreasing potential herniation [8]. Another method that has been documented by Hirahara et al. suggests the placement of the residual greater omentum as a barrier between the Roux limb and the transverse mesocolon [9]. Nonetheless, both methods are burdened by increasing operational time and the limitation of laparoscopic view, which in some cases, ends up with an untight closure.

An article published in 2020 recommended a straightforward and effective approach to minimizing Peterson’s hernia while saving time on defect closure [10]. They suggested that by fixing the Roux limb onto the duodenal stump, the space behind it gets narrower and Peterson's opening angle changes. The result of such reconstruction significantly restricts the mobility of the Roux limb and tightly sticks both the mesentery of the Roux limb and the transverse mesocolon. Finally, we recommend more extensive research on this topic that aims at finding new surgical techniques that help in the prevention of such complications.

## Conclusions

Petersen’s hernia is a rare complication after Roux-en-Y gastric bypass surgery. High clinical suspicion is required for the diagnosis, especially for those presenting with abdominal pain that resembles small bowel obstruction postoperatively, to ensure appropriate management and reduce morbidity and mortality. Many studies have discussed surgical techniques that help in the prevention of such complications. Our research highlights a gap in the literature concerning the impact of omental defect closure in Petersen's hernia cases. We recommend more extensive research on a larger population. 
